# A systematic meta-analytic review of evidence for the effectiveness of the ‘Fast ForWord’ language intervention program

**DOI:** 10.1111/j.1469-7610.2010.02329.x

**Published:** 2011-03

**Authors:** Gemma K Strong, Carole J Torgerson, David Torgerson, Charles Hulme

**Affiliations:** 1 University of YorkUK; 2 University of BirminghamUK

**Keywords:** Language impairment, reading difficulties, auditory processing, Fast ForWord

## Abstract

**Background:**

Fast ForWord is a suite of computer-based language intervention programs designed to improve children's reading and oral language skills. The programs are based on the hypothesis that oral language difficulties often arise from a rapid auditory temporal processing deficit that compromises the development of phonological representations.

**Methods:**

A systematic review was designed, undertaken and reported using items from the PRISMA statement. A literature search was conducted using the terms ‘Fast ForWord’ ‘Fast For Word’ ‘Fastforword’ with no restriction on dates of publication. Following screening of (a) titles and abstracts and (b) full papers, using pre-established inclusion and exclusion criteria, six papers were identified as meeting the criteria for inclusion (randomised controlled trial (RCT) or matched group comparison studies with baseline equivalence published in refereed journals). Data extraction and analyses were carried out on reading and language outcome measures comparing the Fast ForWord intervention groups to both active and untreated control groups.

**Results:**

Meta-analyses indicated that there was no significant effect of Fast ForWord on any outcome measure in comparison to active or untreated control groups.

**Conclusions:**

There is no evidence from the analysis carried out that Fast ForWord is effective as a treatment for children's oral language or reading difficulties.

Fast ForWord is a suite of computer-based intervention programs designed to improve oral language and literacy skills in children with language learning weaknesses. The programs developed from a theory that claims language and literacy learning difficulties in children may arise from impairments in rapid auditory temporal processing skills (Tallal & Piercy, 1973; Tallal, 1980; Reed, 1989; Tallal, Miller, & Fitch, 1993; Tallal et al., 1996; Tallal, 2000). A corollary of this theory is that, given neuroplasticity, appropriate training can lead to lasting improvements in underlying neural systems and concomitant improvements in children's language and reading skills (Merzenich & Jenkins, 1998).

Early studies used a precursor to Fast ForWord on small groups of children with specific language impairment (SLI). Two linked papers by Tallal et al. (1996) and Merzenich et al. (1996) reported data from two small-scale studies. In the first study, which lacked a control group, it was reported that 7 children with language impairments showed improvements on measures of language skills after just 4 weeks' training with a computer-based program using acoustically modified speech. A second, small-scale non-randomised study of 22 children, described in the same articles, compared the effectiveness of an equivalent training program either with, or without, the acoustically modified speech component. It was claimed that the children in the acoustically modified speech group made greater gains on measures of language ability, although these gains would not be reliable based on a conventional (2-tailed) statistical test. We believe that the design limitations (very small sample sizes, and only the second study included a control group but did not use random assignment) and the absence of statistically reliable effects (the effects were reliable on a 1-tailed test only in the second study) preclude any strong claims for the effectiveness of Fast ForWord from these studies.

Based on these early studies, Fast ForWord was launched as a commercial product by the Scientific Learning Corporation, and it is claimed that the program results in a ‘wide range of improved critical language and reading skills’ (see http://www.scilearnglobal.com/the-fast-forword-program/). Fast ForWord is a suite of computer programs that contain language-based audiovisual games designed around themes intended to engage children aged between 4 and 14 years with language difficulties (Scientific Learning Corporation, 1999; http://www.scilearnglobal.com/; http://en.wikipedia.org/wiki/Fast_Forword). The games are adaptive and contain speech that is acoustically modified by a two-stage processing algorithm (Nagarajan et al., 1998) and adapt with the child's progress, gradually decreasing modification. The program also incorporates other language training elements, akin to those used by speech and language therapists, in order to ‘cross-train’ many different skills at the same time (Tallal, 2000).

Fast ForWord was launched commercially in 1997, and is now used in many schools and clinics in the USA, Canada and Australia as well as in the UK and other countries. In a scope of use study by the What Works Clearinghouse (2007) it was estimated that Fast ForWord has been used by over 570,000 children in more than 3,700 schools in the US, with the Scientific Learning Corporation (1999) claiming language gains of 1½ to 2 years in a training period of only 4 to 8 weeks. However, there is much controversy over the effectiveness of Fast ForWord (Cirrin & Gillam, 2008). Many of the claims which the Scientific Learning Corporation make appear to be based on findings from their privately conducted and non-peer-reviewed studies (Scientific Learning Corporation, 1999, 2003) rather than independent studies published in peer-reviewed journals. Moreover, it is still unclear as to whether the improvements observed in these studies are directly related to the acoustically modified speech component of the program.

A scoping review, carried out as background to the present review, revealed four existing systematic reviews dealing with the effectiveness of the Fast ForWord program. The first review was a meta-analysis of the effectiveness of Fast ForWord on academic performance in general (Sisson, 2009). The second reviewed general language interventions for children with spoken language disorders (Cirrin & Gillam, 2008); two reviews, conducted by the What Works Clearinghouse, were intervention effectiveness reports looking at the effects of Fast ForWord on beginner readers and Fast ForWord-Language on English language learners (What Works Clearinghouse, 2007, 2006).

Sisson (2009) carried out a systematic review of studies measuring the efficacy of Fast ForWord and located 31 studies which met inclusion criteria. Effect sizes were computed across many areas of academic skill; however, Fast ForWord was found to have no particular effect on any of the skills analysed. The computed mean effect size was found to be small and pooled effect sizes for each skill were also subject to much variability, suggesting that there are no significant effects of Fast ForWord on academic performance.

Cirrin and Gillam (2008) reviewed studies of language intervention practices published since 1985 which involved school-aged children who were diagnosed with spoken language disorders. A search of electronic databases and a hand search of other sources located 21 studies that the authors judged to have used suitably stringent procedures for evaluating general language interventions; of these, five studies involved Fast ForWord. The conclusion drawn from this review is that programs such as Fast ForWord are ‘neither necessary nor sufficient to induce significant changes in processing or expressive and receptive language skills’ (Cirrin & Gillam, 2008, p. S129).

In a report on the effects of Fast ForWord on beginner readers (kindergarten to third grade) published by the What Works Clearinghouse (2007), five studies were located which met their standards and an additional study was included with reservations. Effectiveness was assessed by outcome measures of phonic reading skills (phonological awareness, phonics and letter knowledge) and comprehension. This report concludes that there are positive effects of Fast ForWord on phonic reading skills but mixed effects on comprehension outcomes. However, the procedures used and the conclusions drawn from this review have been roundly criticised by McArthur (2008); in particular, McArthur argued that this review was largely based on unpublished studies conducted by the Scientific Learning Corporation and failed to include a key study that was published in a peer-reviewed journal.

The What Works Clearinghouse (2006) intervention report for Fast ForWord Language on English language learners reviewed one paper which met evidence standards and one which met them with reservations. These studies focused on phonological awareness, reading achievement and English language development outcome measures in children between kindergarten and sixth grade. The conclusions of this review are that Fast ForWord Language could have a positive effect on English language development but no apparent influence on reading achievement.

The evidence from existing reviews of the effectiveness of the Fast ForWord program appears unpromising. Given the fact that this program is in such widespread use it seems a matter of some importance and urgency to establish whether the program can be deemed to be effective, and if so, for which skills. With this aim in mind, this paper reports a systematic meta-analytic review of evidence for the effectiveness of the Fast ForWord program.

In this paper, we report a meta-analysis of all the studies of Fast ForWord that we could identify that have used an appropriate design (see below for details). We assess the effects of the program on the four critical areas that have been most studied and that are of clear practical and clinical relevance (standardised measures of Single Word Reading, Passage Reading Comprehension, Receptive Vocabulary and Expressive Vocabulary).

## Method

To ensure the rigour of our systematic meta-analytic review, we designed and reported it according to the 27 items from the PRISMA statement (an internationally recognised method for reporting systematic reviews that ensures they are reported to the highest methodological standards, see http://www.prisma-statement.org). To conduct an unbiased and inclusive review, inclusion criteria were predefined in a Protocol or plan of the review, and focused on the substantive relevance and quality of experimental procedure.

### Electronic searching

The databases PsycINFO, Social Policy and Practice, Applied Social Sciences Index and Abstracts, Educational Resources Information Center, CSA Linguistics and Language Behaviour Abstracts, Social Sciences Citation Index, Arts and Humanities Citation Index, and Conference Proceedings Citation Index-Social Science and Humanities were searched. The time period was left open for these searches. The keywords ‘Fast ForWord’ were used together with the variations ‘Fastforword’ and ‘Fast For Word’. See Appendix B (online) for details of the search strategies.

### Citation searching

A bibliographic search was also conducted of the previous systematic reviews to identify any potentially relevant papers not identified through the electronic searching.

### Inclusion/Exclusion criteria

The inclusion criteria were set out in the systematic review Protocol (Appendix A, online) written before the search was initiated. To be included in the review, studies had to be randomised controlled trials or quasi-experiments with a treatment group taking part in the Fast ForWord intervention and at least one other group receiving either no intervention or an alternative treatment. The groups had to be equivalent at baseline after being either randomly assigned or matched. We adopted these criteria for inclusion, because we believe that these designs are the only ones that permit well-founded conclusions about the effectiveness of an intervention. Measures used to assess language abilities in trials had to be standardised tests of reading or oral language. Because the program is produced only in English, only studies on English-speaking participants were included. Participants could be of any age and of any learner characteristics. Only papers published in peer-reviewed journals were included. It is acknowledged that, by using this restriction, a potential source of publication bias could have been introduced which could produce an exaggerated effect of the program.

### Abstract screening

The titles and abstracts of papers located were screened against the inclusion/exclusion criteria. This first stage of screening was carried out by two independent moderators (GS and CT) who screened and then met to compare their results; any disagreement/uncertainty was discussed and arbitrated by a third independent reviewer (CH).

### Screening of full papers

At the second stage of screening, the full papers were screened against the stipulated inclusion criteria. This procedure was again conducted by two independent reviewers (GS and CT) and any discrepancies were resolved by calling upon a third independent reviewer (CH).

### Data extraction

From included papers details of study design, participants, experimental and control conditions, quantified outcome measures and items relating to study quality were extracted and compiled in a standard format. Data were extracted by two reviewers working independently (GS and CT), who then resolved any disagreements. If necessary, a third reviewer was called on to arbitrate (CH).

The outcome measures to be analysed were pre-specified before extraction of numerical data. This review sought to look at the effects of the intervention on both reading and language measures, and the measures selected reflected this. For the reading element a maximum of two outcome measures were included: single word reading and passage reading comprehension, the former being the primary measure. For language, a maximum of two outcome measures were extracted, one measuring receptive vocabulary (primary measure) and the second measuring expressive vocabulary. The measures had to be standardised tests. The quantified outcomes were extracted by two reviewers working independently (GS and CT), with a third reviewer called on to resolve any discrepancies (DT).

We also quality appraised each study using a risk of bias assessment tool based on the CONSORT statement (Altman et al., 2001). We included those five items which, if not followed, are most likely to introduce a potential source of bias. Quality judgements were made by two reviewers working independently, who then met to resolve any discrepancies (CT and DT).

Data were extracted and expressed in tabular form (see Tables 1 and 2 and Table 3 online).

**Table 1:** Descriptive data from included studies

Citation	Participants	Design and setting	Intervention treatment	Control treatment	Outcome measures
Borman, G.D., Benson, J.G., & Overman, L. (2009). A randomised field trial of the Fast ForWord language computer-based training program. *Educational Evaluation and Policy Analysis, 31*, 82–106.	*Number:* 415 children *Age:* 141 2nd grade; 274 7th grade *Inclusion characteristics:* Score below norm (50th percentile) on total reading (CTBS/5). Tendency to have ‘below-average’ language skills. Predominantly African American, low SES.	*Design:* Individually randomised trial. Randomised allocation within each school and within each of the grades. 11 randomisation blocks. Groups roughly equivalent baseline. Grade matched. *Setting:* 8 schools within Baltimore City Public School System - 2 elementary; 3 middle; 3 elementary-middle.	*Intervention:* Fast ForWord during pullout program during school day. *Duration:* Elementary recommended 100 mins per day, 5 days a week for minimum of 20 days. Middle & high school recommended 90 mins per day, 5 days a week for min of 20 days.	Non-literacy instruction or participated in special activities and classes (e.g., art, gym).	Achievement on CTBS/5 (form A) – reading comprehension and language skills. Language and speech skills observational survey completed by teacher.
Cohen, W., Hodson, A., O'Hare, A., Boyle, J., Durrani, T., McCartney, E., et al. (2005). Effects of computer-based intervention through acoustically modified speech (Fast ForWord) in severe mixed receptive-expressive language impairment: Outcomes from a randomised controlled trial. *Journal of Speech, Language and Hearing Research, 48*, 715–729.	*Number:* 77 children *Age:* 6–10 years *Inclusion characteristics:* Referred by SLPs and paediatricians. Diagnosis of receptive SLI. Absence of neurological deficits, pervasive developmental disorders. Normal hearing sensitivity. Nonverbal IQ > 80 (BAS II) or Raven's Coloured Progressive Matrices. Receptive language score <1.3 SD (CELF). Access to landline telephone service at home for internet linkup with FFW required.	*Design:* Randomised controlled trial. Individual randomisation by centre into three groups. No significant difference in baseline measures or age between groups. Equivalence of groups. *Setting:* Home-based.	*Intervention:* Fast ForWord intervention at home. *Duration:* 6 weeks. Days 1–3: 60 minutes; 4–5: 80 minutes; 6+: 100 minutes per day.	*Active control:* Computer based activities designed to promote language as a control for computer games exposure. Encouraged to play 3 packages for 30 mins per day for 5 days a week *Developmental control: N*o additional study intervention. All maintain regular speech language therapy and school regime.	CELF – Receptive, expressive and total language standard score; TOLD – age variation in picture vocabulary and grammatical understanding; PhAB – standard scores on alliteration, rhyme and spoonerisms; BAS II Reading Scale – standard score; Bus Story Test – age variation of information, sentence length and number of subordinate clauses.
Gillam, R.B., Loeb, D.F., Hoffman, L.M.. Bohman, T., Champlin, C.A., Thibodeau, L., et al. (2008). The efficacy of Fast ForWord language intervention in school-age children with language impairment: A randomised controlled trial. *Journal of Speech, Language, and Hearing Research, 51*, 97–119.	*Number:* 216 children *Age:* 6–9 years *Inclusion characteristics:* Language impaired. Parents informed of study and volunteered their children for participation. Score between 75 and 125 on Matrices subtest of Kaufman Brief Intelligence Test. Standard score ≤ 81 on two or more clusters of Test of Language Development – Primary, 3rd edition. No hearing, visual, neurological or oral-structural impairment, emotional or social disorders. No history of 3 or more episodes of otitis media in last 12 months, focal brain lesions, traumatic brain injury, cerebral palsy, seizure disorder, severely impaired reciprocal social interaction or severely restricted activities in DSM-IV criteria for autism. No use of 8 or more hours of other language intervention software or enrolment in other language intervention programmes.	*Design:* Individual random assignment to 4 groups, stratified by treatment site and socio-economic status. *Setting:* Summer intervention program at three treatment sites.	*Intervention:* Fast ForWord Language. *Duration:* 1hr 40 mins per day, 5 days per week for 6 weeks.	*Active control:* Computer Assisted Language Intervention (CALI) *Developmental control:* Academic enrichment (educational computer programs) *Other control:* individualised language intervention (ILI – carried out by SLP.)	Comprehensive Assessment of Spoken Language (CASL) – standardised test of receptive and expressive language. Backward masking. Token test for children. Blending words subtest of Comprehensive Test of Phonological Processing (CTOPP).
Given, B.K., Wasserman, J.D., Chari, S.A., Beattie, K., & Eden, G.F. (2008). A randomised, controlled study of computer-based intervention in middle school struggling readers. *Brain and Language, 106*, 83-97.	*Number:* 65 children *Age:* Middle school (mean age = 12.53 years) *Inclusion characteristics:* Referred due to limited reading progress. Autistic and emotionally disturbed eliminated. Normal auditory acuity and normal/corrected vision. DOB, IQ equivalent scores, reading scores and special education eligibility were reviewed.	*Design*: 25 participants opted and qualified for brain imaging individually randomly assigned to groups. Remaining participants then randomly assigned to 5 groups. *Setting:* 3 middle schools in three mid-Atlantic school divisions.	*Intervention:* Two phases of Fast ForWord intervention. *Duration:* 88 minutes per day, in 2 sessions, 5 days per week.	*Active control*: Success Maker intervention program. *Developmental control:* regular curriculum. *Other Controls:* Crossover interventions – Success Maker followed by Fast ForWord; Fast ForWord followed by Success Maker.	WJ-R - auditory processing subtest for phonological awareness; Rapid Automatised Naming - phonological retrieval; CELF-3 - Receptive language - concepts and directions, word classes and semantic relationships; CELF-3 - Expressive language - formulated sentences, recalling sentences and sentence assembly; WJ-R - Letter-Word Identification, word attack, passage comprehension; Wide Range Achievement Test - spelling.
Pokorni, J.L., Worthington, C.K., & Jamison, P.J. (2004). Phonological Awareness Intervention: Comparison of Fast ForWord, Earobics, and LiPS. *The Journal of Educational Research, 97*, 147–157.	*Number:* 60 children*Age:* 7½–9 years *Inclusion characteristics:* SLP nominated students receiving school-based speech/language services outlined in an individual education plan. Reading more than 1 year below grade level according to school records and teacher reports. English-speaking families. No known hearing impairment. Scored more than 1SD below mean on at least 1 CELF-3 pre-test.	*Design:* Individual random assignment to 3 groups within each geographical area. *Setting:* 20-day summer program within 3 schools acting as intervention sites. Each group in separate rooms.	*Intervention:* Fast ForWord intervention. *Duration:* Three 1-hour interventions per day for 20 days.	*Active control:* Lindamood Phonemic Sequencing program (LiPS) intervention. *Other control:* Earobics intervention program.	Phonological Awareness Test (PAT): phoneme blending, phoneme segmentation; Clinical Evaluation of Language Fundamentals-3 (CELF-3): concepts and directions, recalling sentences, listening to paragraphs; Woodcock Language Proficiency Battery-revised (WLPB-R): letter–word identification, passage comprehension, word attack, spelling.
Rouse, C.E., & Krueger, A.B. (2004). Putting computerised instruction to the test: A randomised evaluation of a ‘scientifically based’ reading program. *Economics of Education Review, 23*, 323–338.	*Number:* 454 children *Age:* Grades 3–6 *Inclusion characteristics:* Bottom 20% (state-wide) or significantly below grade level on the state's standardised reading test. Principal assessment of whether children could sit through 90–100 mins of computer activity.	*Design:* Individual random allocation to treatment or control group, within each school and grade. *Setting:* Four schools within an urban school district with below average test scores.	*Intervention:* pull-out program of Fast ForWord intervention. Missing homeroom, math, science, language arts and specials or before/after school. *Duration:* Fast ForWord: days 1–3: 60 minutes; 4–5: 80 minutes; 6+: 100 minutes per day. 90 minutes per day for Fast ForWord middle school and language-to-reading.	No intervention	Reading edge- accelerated mode; CELF-3: Receptive portion (CELF-3-RP): concepts and directions, word classes, semantic relationships, listening to paragraphs; Success for All assessments (SFA).

### Meta-analyses

We undertook a series of meta-analyses combining the results from the studies for each of the outcome measures for reading and language (details below).

## Results

### Searching and screening

The initial electronic search located 130 studies containing the key words (‘Fast ForWord’ ‘Fastforword’ and ‘Fast For Word’). Once duplicates were removed this was reduced to 79 potentially relevant studies.

Cohen's kappa coefficient was calculated to assess concordance between the two independent screenings of titles and abstracts of the 130 studies. Highly significant inter-rater reliability was found: κ = .84. This high level of agreement, combined with the double screening procedures, ensured reliability of inclusion decisions at the first stage. Four studies were not initially agreed upon; following discussion between two raters (GS and CT) two were excluded due to lack of a control group and the remaining two were sent to a third rater (CH) for arbitration. These were also excluded on the grounds of (a) non-matched experimental groups and (b) poor randomisation and inadequate sample size: they were judged to be of insufficiently high quality to be included. Thirteen studies were included at the first stage. No further studies were located during the citation search.

At the second stage of screening (screening of the 13 full papers) a further seven papers were excluded. Two were excluded due to lack of baseline equivalence (Troia & Whitney, 2003; Hook, Macaruso, & Jones, 2001), four were excluded as they did not use the commercially available Fast ForWord program (Bishop, Adams, & Rosen, 2006; Bishop, Adams, Lehtonen, & Rosen, 2005; Wren & Roulstone, 2008; Ukrainetz, Ross, & Harm, 2009) and one was excluded because it was a corrigendum to a study included within the review (Given, Wasserman, Chari, Beattie, & Eden, 2009). The corrigendum was noted in the original paper (Given, Wasserman, Chari, Beattie, & Eden, 2008).

After first and second stages of screening, six papers remained for data extraction and quality assurance (quality judgements focused primarily on attrition and participant fidelity to the experimental procedure).

### Characteristics of the studies

Characteristics of all the included studies (bibliographic details, study design, participants, design, experimental and control groups, outcome measures and quality indices) are shown in Table 1 and Table 3 (online).

All six included studies were individually randomised controlled trials, with sample sizes ranging from 60 to 454. The ages of the participants ranged from about 6 to about 11 years, with the exception of the study by Given et al. (2008), where the children were slightly older, with a mean age of 12.5 years. The trials were undertaken in school settings (3 trials), home-based (1 trial) or in summer school settings (2 trials).

Table 2 presents the quantified outcomes for all included studies for comparisons involving both active and non-active controls, and for reading and language primary and secondary outcomes. Table 3 (online) presents results of quality appraisal of the six studies.

**Table 2:** Data extraction of outcome measure

Means for intervention and non-active controls (standard deviations given in parentheses)						
Study		*n*	Single Word Reading	Passage Reading	Receptive Vocabulary	Expressive Vocabulary
Given et al.	Intervention	12	1.58 (7.56)	2.17 (6.18)	3.42 (9.69)	5.25 (10.60)
	Control	13	3.08 (3.80)	–.92 (8.86)	4.15 (11.19)	9.15 (7.29)
Gillam et al.	Intervention	51	–	22.6 (7.4)	83.0 (13.3)	83.0 (13.3)
	Control	53	–	21.6 (8.8)	82.1 (11.4)	82.1 (11.4)
Rouse & Krueger	Intervention	237	44.57 (24.78)	–	–	–
	Control	217	43.03 (24.0)	–	–	–
Cohen et al.	Intervention	23	89.22 (18.81)	–	72.22 (6.04)	68.35 (5.83)
	Control	27	83.11 (12.01)	–	72.44 (5.77)	68.81 (4.80)

Means for intervention and active controls (standard deviations given in parentheses)						
Study		*n*	Single Word Reading	Passage Reading	Receptive Vocabulary	Expressive Vocabulary
Given et al.	Intervention	12	1.58 (7.56)	2.17 (6.18)	3.42 (9.69)	5.25 (10.60)
	Control	14	.78 (5.58)	4.50 (6.76)	9.07 (11.91)	9.64 (10.82)
Gillam et al.	Intervention	51	–	22.6 (7.4)	83.0 (13.3)	83.0 (13.3)
	Control	53	–	22.3 (8.2)	83.4 (11.7)	83.4 (11.7)
Pokorni et al.	Intervention	20	80.3 (13.6)	86.0 (13.8)	79.0 (32.6)	72.5 (23.6)
	Control	18	87.9 (12.2)	89.6 (8.2)	62.8 (30.4)	62.2 (20.7)
Cohen et al.	Intervention	23	89.22 (18.81)	–	72.22 (6.04)	68.35 (5.83)
	Control	27	84.56 (10.95)	–	72.22 (8.79)	71.26 (9.65)

We applied a high threshold of quality for inclusion in this review, and all studies were of a high quality in the sense that they employed a randomised controlled trial design. However, in terms of the risk of bias assessment tool the studies did vary somewhat, with only two stating that the allocation was concealed and only three stating that outcome ascertainment was undertaken blind to group allocation. Attrition was fairly high in four of the studies, but the studies by Borman, Benson, and Overman (2009) and Rouse and Krueger (2004) noted this factor and undertook both intention-to-treat (ITT) analyses and instrumental variable (IV) or LATE analyses. None of the studies undertook selective outcome reporting. Therefore, although the studies varied in quality, and we were not able to rule out the possibility of bias having been introduced into the individual studies, on balance the risk was judged to be low due to all of the study authors including attempts to limit bias in their individual trials. If outcome data were available in the individual studies in order for us to include these in a meta-analysis, we did so.

### Meta-analyses

The study by Borman et al. (2009) was not included in the meta-analysis due to there being insufficient data in the paper. Efforts were made to obtain data from the authors; however, at the time of submission no response had been received.

Only one Single Word Reading outcome measure was used for the study by Rouse and Krueger (2004) as the levels of attrition were too high (>20%) for other outcome measures to be extracted and included. This study also has only an untreated control group.

Gillam et al. (2008) published a composite score for receptive and expressive vocabulary, so these combined means were entered for both measures of oral language. A primary measure in this study, the Comprehensive Assessment of Spoken Language (Carrow-Woolfolk, 1999), is a standardised test of both expressive and receptive language with strong psychometric properties and high reliability. The composite score was produced by taking the sum of various subtests which were selected specifically for children of differing ages (7–10-year-olds), and although only one subtest was used for 6-year-old children, this was not part of the core composite score.

In the case of Pokorni, Worthington, and Jamison (2004) there was no untreated control group, thus the study was not included in the untreated control meta-analyses. For this study, the ‘LiPS’ active control was selected rather than the ‘Earobics’ group on the basis that it was stated in the introduction of the paper that this intervention had been used more frequently in published studies and there was no empirical evidence of the effectiveness of Earobics. The group size was also larger for the LiPS intervention.

A series of eight meta-analyses comparing the Fast ForWord intervention group with (a) untreated controls and (b) active controls receiving an alternative treatment on four outcome measures (Single Word Reading, Passage Reading Comprehension, Receptive Language, and Expressive Language) were conducted using Arcus Quickstat. Standardised effect sizes (Cohen's *d*) for all pre-specified outcome measures in each study were computed by dividing the mean difference between groups at post-test by a pooled standard deviation. This was carried out for each of the four outcome measures and for comparisons involving both active and untreated control groups. The effect sizes were then pooled across studies for each domain. Studies which did not contain one of the measures were excluded from that specific analysis.

The results of the meta-analyses we conducted are remarkably clear. For the 4 analyses of Fast ForWord compared to untreated control groups, the pooled effect size was .079 (95% CI −.09 to .25), .17 (95% CI −.17 to .52) for passage comprehension, .01 (95% CI −.25 to .28) for receptive language and −.04 (95%−.33 to .25) for expressive language. For comparisons with the treated control groups the equivalent pooled effect sizes were −.026 (95% CI −.40 to .35), −.10 (95% CI −.40 to .21) for passage comprehension, .02 (95% CI −.27 to .31) for receptive language and −.06 (95%−.33 to .20) for expressive language. None of the 8 pooled effect sizes were reliably different from zero, and 4 of the effect sizes were actually negative (indicating worse performance in the Fast ForWord treatment group than the control group). Thus from the studies we have identified and analysed here there is no convincing evidence that Fast ForWord is effective in improving children's single word reading, passage reading comprehension, receptive language or expressive language skills.

## Discussion

Fast ForWord is a commercially distributed suite of computer-based intervention programs that is designed to remediate oral language and literacy difficulties in children. There is no evidence from this review that the program is effective as a treatment for children's reading or expressive or receptive vocabulary weaknesses.

### Relationship to previous studies

The negative results from this meta-analysis actually align well with the results from three other training studies that we did not include in this review.

The first study, by Borman et al. (2009), could not be included in our meta-analysis because the published paper does not give the data necessary for the computation of effect sizes. This study is a high-quality randomised field trial that evaluated the effectiveness of the Fast ForWord program in eight schools, with random assignment of children within schools to the treatment or no-treatment control groups. The study appears to have been conducted in collaboration with the Scientific Learning Corporation, the developers of the Fast ForWord program. The findings reported in this paper are nevertheless entirely consistent with the pattern obtained from our meta-analysis. In an analysis based on 107 children the effect of the Fast ForWord treatment on Language outcome (*d* = +.08) was positive but non-significant and on Reading Comprehension outcome (*d* = −.07) was negative but non-significant.

There are two other studies that we did not include because they did not use the commercially available Fast ForWord program. These two studies used the same sample of children who were given both forms of intervention at the same time. Bishop, Adams, Lehtonen, and Rosen (2005) reported a small-scale study of a computerised program for training spelling skills given to children with language impairments. In this program the children attempted to spell words presented as pictures on a computer screen and when they could not do so they were provided with spoken prompts (the next sound from the word that was to be written) using either natural or acoustically modified speech. The modified speech condition used the same computer algorithm used in Fast ForWord. Children in the study succeeded in learning some of the trained words but the training did not appear to generalise since the trained children did not make any greater gains on a standardised spelling test than an untrained control group. Most significantly, in relation to the current review, the trend was for children trained with the acoustically modified speech to make less progress than those trained with natural speech, and this was true even for children who were shown, using experimental measures, to have auditory temporal processing impairments (for whom the modified speech should have been effective).

Similarly, Bishop, Adams, and Rosen (2006) used a computerised grammatical training program with children with receptive language impairments. In this study they compared the effectiveness of acoustically modified speech (using the same computer algorithm as Fast ForWord) with a normal speech condition. The two trained groups did not differ from an untrained control group on measures of auditory language skills; furthermore, once again there was no sign that the acoustically modified speech group did better than the children in the unmodified speech condition.

In summary, these two small-scale, well-controlled studies also failed to find evidence for the effectiveness of using acoustically modified speech (comparable to that used in Fast ForWord) for training the language and literacy skills of children with language impairments. Similarly, the Borman et al. (2009) study failed to find evidence for the effectiveness of the Fast ForWord program from a randomised field trial.

### Practical and theoretical implications

This review clearly provides no support for the effectiveness of the Fast ForWord program as a treatment for children with language and literacy impairments. We certainly need to be cautious, insofar as null results can never demonstrate the absence of an effect. However, from an applied perspective the current review suggests that the Fast ForWord program cannot be endorsed as a suitable treatment for children with oral language or reading difficulties. In our opinion the studies reviewed here were adequate methodologically, and had reasonable to large sample sizes; therefore, even if the effects from the Fast ForWord program were small, the studies reviewed should have been able to detect them.

The finding that Fast ForWord is not effective in remediating children's language difficulties might at first appear to refute the theory underlying the program (that language learning problems are caused by a rapid auditory temporal processing deficit). However, in order to refute the theory that language learning problems in some children may be caused by a rapid auditory temporal processing deficit, it would be necessary to show that suitable training for such children can produce substantial improvements in rapid auditory processing skills, without producing concomitant improvements in language skills. Only one study included in this review (Gillam et al., 2008) included a measure of rapid auditory temporal processing (auditory backward masking). This study found that Fast ForWord was no more effective in improving this measure than either of two alternative treatments that did not involve acoustically modified speech. These findings certainly throw considerable doubt on the claims made by the developers of Fast ForWord that suitable training can remediate rapid auditory processing problems and in turn improve the language skills of children with language impairments (cf. Tallal et al., 1996).

In one other relevant experimental study, McArthur, Ellis, Atkinson, and Coltheart (2008) studied groups of children with SLI and reading disorders and found that a large proportion of these children showed abnormalities on a range of auditory psychophysical measures (including measures of rapid auditory temporal processing). The children with auditory processing deficits were given intensive training over 6 weeks targeting their auditory processing weaknesses. The training was successful in remediating these children's basic auditory processing weaknesses, but this did not transfer to effects on measures of oral language or spelling. Thus in this study training was effective in remediating auditory processing skills, but had no transfer effects to literacy or language measures. It remains important for future studies in this area to establish whether (a) suitable training programs can be developed that can produce improvements in children's rapid auditory temporal processing, and (b) if so, whether such improvements are associated with (i.e., mediate) corresponding improvements in language processing skills. Given that these were the explicit aims of the developers of Fast ForWord, the negative evidence from the study by Gillam et al. (2008) and McArthur et al. (2008) is certainly not encouraging.

A full review of other forms of treatment for children with language and reading difficulties is beyond the scope of this paper, but it may worth considering briefly how the results reviewed here concerning Fast ForWord compare with the results of studies using different (more conventional) forms of therapy.

If we consider interventions for children with oral language difficulties there are not large numbers of randomised controlled trials, and arguably some of the ones that do exist are of low methodological quality (see, Laws, Garrett, & Nye, 2004). Nevertheless, the evidence from the meta-analysis of therapy studies by Laws et al. does show that one of the primary outcome measures considered here (Expressive Vocabulary) is associated with large effect sizes for standardised measures of expressive vocabulary (*d* = .98; *n* = 74; 95% CI −.5–2.56), measures of the number of words used in expressive language samples (*d* = 1.08; *n* = 82; 95% CI .61–1.55), and parental ratings of vocabulary usage (*d* = .89; *n* = 136; 95% CI .21–1.56). Thus there is reasonable evidence that conventional forms of therapy (which include direct teaching of vocabulary) are effective in improving vocabulary knowledge.

There are many studies of the effectiveness of remedial teaching methods for children with reading (decoding) difficulties. One of the primary outcome measures for the present meta-analysis was single word reading and there is very good evidence that difficulties in developing accurate word reading skills can be effectively ameliorated with phonically based reading instruction coupled with phoneme awareness training. Bus and van IJzendoorn (1999) and the National Reading Panel (NRP) in the US (National Institute of Child Health and Human Development, 2000) both reported meta-analyses of relevant studies. Bus and van IJzendoorn reported that phonological awareness training improved reading skills (*d* = .44) and this effect did not differ significantly for typical versus struggling readers (*d* = .40 vs. .60). In line with this, the NRP meta-analysis (National Institute of Child Health and Human Development, 2000) also found that phoneme awareness training produced a moderate and reliable effect on reading outcomes (*d* = .53).

In summary there is relatively good evidence that conventional forms of therapy have larger effects on some key language and reading outcomes than those reported in this meta-analytic review of Fast ForWord.

## Conclusions

We believe that the pattern shown by our analyses is clear and consistent: whether comparing Fast ForWord with untreated or alternative treatment control groups, we found no sign of a reliable effect of treatment in any analysis. There is no evidence from this review that Fast ForWord is effective as a treatment for children's reading or expressive or receptive vocabulary weaknesses. In contrast, evidence suggests that conventional forms of therapy can effect modest but reliable improvements in these skills.

**Key points**

- We have conducted a systematic meta-analytic review of the Fast ForWord language intervention program.
- There is no evidence from the published studies reviewed that Fast ForWord is effective as a treatment for reading difficulties or receptive or expressive vocabulary difficulties in children.
- Our findings cast strong doubt on the clinical effectiveness of the Fast ForWord program.
- Auditory temporal processing skills, the construct that theoretically is supposed to mediate the putative effects of Fast ForWord training on language outcomes, were only measured in one study reviewed here, and did not appear to improve as a result of Fast ForWord training.

## Supplementary material

The following supplementary material is available for this article:

**Table 3** Risk of bias assessment of included studies.

**Appendix A.** Protocol.

**Appendix B.** Search strategy (Word document)

This material is available as part of the online article from:

http://onlinelibrary.wiley.com/doi/abs/10.1111/j.1469-7610.2010.02329.x
