# Supplementary material for: A systematic meta-analytic review of evidence for the effectiveness of the ‘Fast ForWord’ language intervention program
Source: J Child Psychol Psychiatry. 2011 Mar;52(3):224–35. doi: 10.1111/j.1469-7610.2010.02329.x (PMC3061204; doi:10.1111/j.1469-7610.2010.02329.x)
Supplement: Supplementary file 1 [file jcpp0052-0224-SD1.doc]

**Table 3** Risk of bias assessment of included studies

|  | Borman | Cohen | Gillam | Given | Pokorni | Rouse |
| --- | --- | --- | --- | --- | --- | --- |
| Sequence generation | N | N | N | N | N | N |
| Allocation concealment | NS | Y | Y | NS | NS | NS |
| Blinding | NS | Y | NS | Y | Y | NS |
| Incomplete outcome data | Y | N | N | Y | Y | Y |
| Selective outcome reporting | N | N | N | N | N | N |
| Other | ITT analysis and LATE analysis |  |  |  |  | ITT and IV analysis |

[app]**Appendix A** Protocol

[a]**Title**

[txt]A systematic review and meta-analysis of randomised controlled trials and quasi-experiments investigating the effectiveness of the computer-based language intervention program Fast ForWord on children’s reading and/or oral language skills.

[a]**Context and conceptual issues**

[txt]Research into the aetiology of language impairments has suggested that a temporal auditory processing deficit is at the centre of the poor development of language abilities (Tallal et al., 1996; Merzenich et al., 1996). It has been found that those with specific language impairments require a much longer time period to integrate information in comparison to those with normal development, meaning that they are less able to discriminate speech (Tallal, 2000; Llinas, Ribary, & Tallal, 1998). The outcome of this is that development of phonological processing and grammatical morphology are impaired, leading to language difficulties spanning speaking, writing and reading (Tallal, 2000). It has been proposed that training the processing of rapid acoustic stimuli could ameliorate these problems, leading to improved linguistic skills (Tallal et al., 1996; Merzenich et al., 1996). This has led to the production of Fast ForWord, a set of adaptive, computer-based, training exercises using acoustically modified speech gradually progressing and improving the child’s acoustic processing rate. It is thought that these improvements will increase ability in language tasks. Fast ForWord is now widely commercially available and is used in many schools and clinics in the USA and Canada, claiming that children with language problems can make language gains equivalent to 1½–2 years after training for only 4–8 weeks (Scientific Learning Corporation, 1999); however, there is much controversy concerning the effectiveness of this program.

[a]**Scoping review**

[txt]A scoping review revealed four systematic reviews. The first reviewed general language interventions for children with spoken language disorders (Cirrin & Gillam, 2008), two reviews were intervention effectiveness reports looking at the effects of Fast ForWord on beginner readers and Fast ForWord-Language on English language learners (What Works Clearinghouse, 2007, 2006). The fourth review was a meta-analysis of the effectiveness of Fast ForWord on academic performance in general.

Cirrin and Gillam (2008) reviewed studies of language intervention practices published since 1985 which involved school-aged children who were diagnosed with spoken language disorders. A search of electronic databases and a hand search of other sources located 21 studies with suitably stringent procedures concerning general language interventions; of these, five studies involved Fast ForWord. The conclusion drawn from this review is that programs such as Fast ForWord are ‘neither necessary nor sufficient to induce significant changes in processing or expressive and receptive language skills’ (Cirrin & Gillam, 2008, p. S129).

In an intervention report of the effects of Fast ForWord on beginner readers (kindergarten to third grade) published by the What Works Clearinghouse (2007), five studies were located which met WWC evidence standards and an additional one was included with reservations. Effectiveness of student outcome measures on alphabetics and comprehension was examined. Alphabetics encompasses phonological awareness, phonics and letter knowledge. This report concluded that there are positive effects of Fast ForWord on alphabetics but mixed effects on comprehension outcomes.

The What Works Clearinghouse (2006) intervention report for Fast ForWord-Language on English language learners reviewed one paper which met evidence standards and one which met them with reservations. These studies focused on phonological awareness skill, reading achievement and English language development outcome measures in children between kindergarten and sixth grade. The conclusions of this review are that Fast ForWord-Language could have a positive effect on English language development but no apparent influence on reading achievement.

Sisson (2009) carried out a systematic review of studies measuring the efficacy of Fast ForWord and located 31 studies which met the inclusion criteria. Effect sizes were computed across many areas of academic skill; however Fast ForWord was found to have no particular effect on any of the skills analysed. The computed mean effect size was found to be small and pooled effect sizes for each skill were also subject to much variability, thus suggesting that few assertions can be made in confidence and moreover that there appears to be no significant effect of Fast ForWord on academic abilities. Therefore Sisson suggests that the auditory temporal processing hypothesis of SLI also needs to be reviewed.

[a]**Aim of review**

[txt]The aim is to review the evidence in order to assess the effectiveness of the Fast ForWord intervention program on improving children’s reading and oral language skills.

[a]**Research question**

[txt]The research question is: is Fast ForWord an effective language intervention program for children? Also, does it improve the language skills as proposed by the foundational research and to the extent purported?

[a]**Search strategy**

[b]*Electronic searching*

[txt]The databases PsycINFO; Social Policy and Practice; Applied Social Sciences Index and Abstracts (ASSIA); Educational Resources Information Center (ERIC); CSA Linguistics and Language Behaviour Abstracts; Social Sciences Citation Index; Arts and Humanities Citation Index; and Conference Proceedings Citation Index-Social Science and Humanities will be searched. The time period will be open for these searches as it is a recent innovation. Due to the very specific nature of this review the only required and most sensitive keywords are ‘Fast ForWord’; however, variations will also be searched for due to differing representations. These will be ‘Fastforword’ and ‘Fast For Word’.

[b]*Citation searching*

[txt]A bibliographic search will also be conducted of the previous systematic reviews to identify any potentially relevant papers not identified through the electronic searching.

[a]**Inclusion/exclusion criteria**

[txt]To be included in the review, studies must comprise randomised controlled trials or quasi-experiments with a treatment group taking part in Fast ForWord intervention and at least one other condition group engaging in either no intervention or an alternative program. Measures used to assess language abilities in trials must be standardised tests of reading or oral language and due to the fact that the program is produced only in English, only studies on English-speaking participants and written in English will be included. Participants can be of any age and with any deficit. Only papers published in peer-reviewed journals will be considered.

[a]**Screening of titles and abstracts**

[txt]The titles and abstracts of papers located will be screened against the inclusion/exclusion criteria. Two independent checkers will review the papers; any uncertainty will be discussed and if necessary passed on to a third independent checker.

[a]**Screening of full papers**

[txt]The full papers will be screened against the inclusion/exclusion criteria. Two independent checkers will initially screen the papers, accurately checking them against the stipulated criteria for inclusion. Should there be any discrepancies a third independent checker will be called upon to confirm a decision.

[a]**Data extraction and analysis**

[txt]From included papers details of participants, experimental groups, intervention practices, language measures, outcomes and quality will be extracted and compiled in a standard format. Tabulation of studies will be assembled and effect sizes of outcomes will be calculated. A standardised effect size of all studies will be computed on a commercially available statistics package by dividing the mean difference between groups by a pooled standard deviation.

[a]**Quality assessment of studies**

[txt]All experimental methods of studies will be assessed for risk of bias, using a risk of bias assessment tool developed from the CONSORT statement (Altman et al., 2001).

[app]**Appendix B** Search strategy

*Searched on OVIDSP:*

PsycINFO (1806 – week 1 2009), Social Policy and Practice (200910)

28 hits

*File – “Ovid Results”*

Search strategy:

1 Fast Forword.ti,ab. (27)

2 Fast For Word.ti,ab. (3)

3 Fastforword.ti,ab. (0)

4 1 or 3 or 2 (28)

*Searched via CSA ILLUMINA:*

ASSIA (1987 – current), ERIC (1966 – Current), CSA Linguistics and

Language Behaviour Abstracts (1973 – current)

50 Hits

*File – “CSA ILLUMINA results”*

Search strategy:

*Search Query #1* (*fast forword*) or *fastforword* or (*fast for word*)

(Copy Query

<http://csaweb112v.csa.com/ids70/p_history.php?id=6&Go_Run&SID=f9q8co67k5glhnn0h7rpgnn184>)

50 Published Works results found in Multiple Databases * + *

[<http://csaweb112v.csa.com/ids70/history.php?expand=6,pubworks&SID=f9q8co67k5glhnn0h7rpgnn184>](http://csaweb112v.csa.com/ids70/history.php?expand=6,pubworks&SID=f9q8co67k5glhnn0h7rpgnn184)

2082 Scholars results found in COS Scholar Universe: Social Science

0 Web Sites results found in Web Resources Related to the Social

Sciences/Humanities

Date Range:

Earliest to 2010

*Searched on ISI Web of Knowledge:*

Social Sciences Citation Index (1956 – Present), Arts and Humanities

Citation Index (1975 – Present), Conference Proceedings Citation Index –

Social Science and Humanities (1990 – Present)

52 hits

*File – “ISI Web of Knowledge results”*

Search Strategy:

Topic=("Fast Forword") OR Title=("Fast Forword") OR Topic=("Fast For

Word") OR Title=("Fast For Word") OR Topic=(Fastforword) OR

Title=(Fastforword)

/Databases=SSCI, A&HCI, CPCI-SSH Timespan=All Years/
